# Control of Morphological Differentiation of *Streptomyces coelicolor* A3(2) by Phosphorylation of MreC and PBP2

**DOI:** 10.1371/journal.pone.0125425

**Published:** 2015-04-30

**Authors:** Nils Ladwig, Mirita Franz-Wachtel, Felix Hezel, Boumediene Soufi, Boris Macek, Wolfgang Wohlleben, Günther Muth

**Affiliations:** 1 Interfakultaeres Institut für Mikrobiologie und Infektionsmedizin Tuebingen IMIT, Mikrobiologie/Biotechnologie, Eberhard Karls Universitaet Tuebingen, Auf der Morgenstelle 28, 72076 Tuebingen, Germany; 2 Proteome Center Tuebingen, Interfakultaeres Institut für Zellbiologie, Eberhard Karls Universitaet Tuebingen, Auf der Morgenstelle 15,72076 Tübingen, Germany; University of Groningen, Groningen Institute for Biomolecular Sciences and Biotechnology, NETHERLANDS

## Abstract

During morphological differentiation of *Streptomyces coelicolor* A3(2), the sporogenic aerial hyphae are transformed into a chain of more than fifty spores in a highly coordinated manner. Synthesis of the thickened spore envelope is directed by the *Streptomyces* spore wall synthesizing complex SSSC which resembles the elongasome of rod-shaped bacteria. The SSSC includes the eukaryotic type serine/threonine protein kinase (eSTPK) PkaI, encoded within a cluster of five independently transcribed eSTPK genes (*SCO4775-4779*). To understand the role of PkaI in spore wall synthesis, we screened a *S*. *coelicolor* genomic library for PkaI interaction partners by bacterial two-hybrid analyses and identified several proteins with a documented role in sporulation. We inactivated *pkaI* and deleted the complete *SCO4775-4779* cluster. Deletion of *pkaI* alone delayed sporulation and produced some aberrant spores. The five-fold mutant NLΔ4775-4779 had a more severe defect and produced 18% aberrant spores affected in the integrity of the spore envelope. Moreover, overbalancing phosphorylation activity by expressing a second copy of any of these kinases caused a similar defect. Following co-expression of *pkaI* with either *mreC* or *pbp2* in *E*. *coli*, phosphorylation of MreC and PBP2 was demonstrated and multiple phosphosites were identified by LC-MS/MS. Our data suggest that elaborate protein phosphorylation controls activity of the SSSC to ensure proper sporulation by suppressing premature cross-wall synthesis.

## Introduction

The peptidoglycan (PG) sacculus, an elastic mesh of linear carbohydrate chains cross-linked by short peptides surrounds the bacterial cell [1,2]. This exoskeleton is the cell shape-maintaining element and allows the cell to withstand alterations in internal turgor due to changing environments. Only few bacterial species developed a life style devoid of a PG sacculus [3]. Whereas Gram-negative bacteria have a PG-monolayer, Gram-positive species have a thick multi-layered cell wall that in addition contains large amounts of covalently bound anionic glycopolymers, called wall teichoic acids (WTA) [4,5]. Despite high conservation of the basic PG structure, various species specific modification including different amino acids in the stem peptide or variations in the interpeptide bridge or O-acetylation occur [6].

A major factor in cell shape determination is the site of PG-synthesis [7]. Cocci incorporate PG mainly at the division plane [8], generating a spherical morphology. Incorporation of PG at the lateral wall in a process called elongation growth [9,10], produces a rod-shaped morphology. A key determinant of elongation growth is the actin-like MreB protein [11], which has been originally suggested to form a dynamic helical filament underneath the surface to direct PG synthesis [12–14]. More recent studies do not support helical filaments and describe MreB patches that passively move along with the putative cell wall extension machinery [15,16]. Whereas Gram-negative bacteria have a single MreB protein, the Gram-positive *Bacillus subtilis* requires three MreB homologues, MreB, Mbl and MreBH, for growth [17]. Depletion of MreB, or its inhibition by the antibiotic A22, results in the loss of rod-shape and eventually cell death [17,18]. MreB forms a lateral wall synthesizing complex with MreC, MreD, the PBP2, RodA, and RodZ [19]. In *B*. *subtilis* also interactions with WTA-synthesizing proteins have been shown [20], suggesting that the lateral wall synthesizing complex might also be involved in the synthesis of WTAs.

Actinobacteria are a group of Gram-positive bacteria with different shapes, including spheres, rods and branched mycelium. Their mode of growth does not depend on Mre-proteins, as demonstrated by the absence of *mre* genes in most genera [21]. Instead they incorporate new PG at the cell poles, dependent on the DivIVA protein [22,23]. In mycelial *Streptomyces*, the hyphal growth at the tip is mediated by the polarisome which consists of the coiled-coil proteins DivIVA, Scy and FilP [24,25]. Although tip growth does not depend on Mre proteins, *Streptomyces* and related actinomycetes contain an *mre* gene cluster consisting of *mreBCD*, *pbp2*, and the *rodA* homolog *sfr* [21,26]. However these genes are not essential but affect synthesis of the thickened spore wall which makes *Streptomyces* spores resistant to detrimental environmental conditions [27,28].

Since the protein-protein interaction pattern of the *S*. *coelicolor* Mre proteins highly resembled the interaction pattern of Mre proteins forming the lateral wall synthesizing complex of rod-shaped bacteria, the term *Streptomyces* spore wall synthesizing complex SSSC was coined [28,29]. Screening of a genomic library for additional components of the SSSC identified SCO4778 (PkaI), encoding a eukaryotic type serine/threonine kinase (eSTPK) as an interaction partner of MreC, MreD, PBP2, and Sfr [28].

For a long time it was thought that signal transduction in bacteria relies only on histidine/aspartate phosphorylation via two-component systems, while protein phosphorylation by eSTPKs is limited to eukaryotes. But with the increasing availability of genomic data, more and more eSTPKs were identified in bacteria. For example, *S*. *coelicolor* contains more than 30 eSTPKs [30,31] and more than 50 eukaryotic-type protein phosphatases [32]. The catalytic domains of many bacterial eSTPKs are linked to additional domains. These domains can be extracellular and often mediate ligand binding or protein-protein interaction. The *M*. *tuberculosis* kinase PknB was shown to become activated by dimerization upon binding of its extracellular PASTA-domain to muropeptides [33,34]. Regulation of proteins involved in cell division and cell wall synthesis by phosphorylation is well documented in different bacteria [35]. Phosphorylation can result in the activation of an enzyme or its inhibition. Whereas activity of the muropeptide ligase MurC is inhibited in *Corynebacterium glutamicum*, when phosphorylated by PknA [36], GlmM of *E*. *coli*, involved in peptidoglycan precursor synthesis is activated by phosphorylation [37].

Phosphorylation of one of the Mre proteins has not been reported yet in any bacterium. Here we show that PkaI is able to multiply phosphorylate coexpressed MreC and PBP2 proteins, providing evidence that sporulation septation and the synthesis of the thickened *Streptomyces* spores wall by the SSSC is controlled by protein phosphorylation.

## Results

### Expression of *pkaI* is down-regulated during morphological differentiation

Interaction of PkaI with several SSSC proteins suggested a role of PkaI in sporulation [28]. PkaI is encoded in a cluster of five putative eSTPK genes, *SCO4775 (pkaH)*, *SCO4776*, *SCO4777 (pkaD)*, *SCO4778 (pkaI)*, *SCO4779 (pkaJ)* in *S*. *coelicolor* M145 [31]; S1A Fig). The genomic organization of the eSTPK genes, with intergenic sequences between 93 bp and 173 bp, indicates that each gene is transcribed separately. This was confirmed by RT-PCR. Primer pairs designed to amplify the intergenic regions of each of these genes did not produce the respective fragments, when cDNA of *S*. *coelicolor* was used as a template (S1B Fig).

To elucidate a possible role in morphological differentiation, we characterized the expression profiles of *pkaI* and the other eSTPK genes at distinct stages of the *S*. *coelicolor* life cyle by semi-quantitative RT-PCR with gene-specific primer pairs (S1C Fig). Whereas expression of *pkaH* could not be detected in any growth phase, *SCO4776* was constitutively expressed throughout the whole life cycle. In contrast, *pkaD*, *pkaI*, and *pkaJ* showed highest transcription after 24 hours when mainly substrate mycelium was formed. During aerial mycelium formation, transcription of all three genes was down-regulated. This indicates that PkaD, PkaI, and PkaJ accomplish their function in hyphal growth before or at the onset of morphological differentiation.

### The *ΔpkaI* mutant NLΔPkaI is affected in proper sporulation

To assess the putative role of the eSTPK PkaI in the SSSC, we generated an in frame deletion mutant of *pkaI* that had no polar effects on the downstream *pkaJ* (S2 Fig). The NLΔPkaI mutant showed normal growth in liquid culture. Only sporulation was slightly retarded on solid agar (S3A Fig). Whereas the parental M145 started to differentiate on SM agar after two days, white aerial mycelium of NLΔPkaI was not visible before the third day of incubation. After seven days, the sporulation of NLΔPkaI was nearly indistinguishable from that of M145. This delay in sporulation corresponded also to the generation of viable spore particles. After three days of incubation, 50 fold less spores could be isolated from a NLΔPkaI culture, compared to M145. After five days of incubation, the spore titers obtained from NLΔPkaI and M145 were similar (S3B Fig).

**Fig 1:**
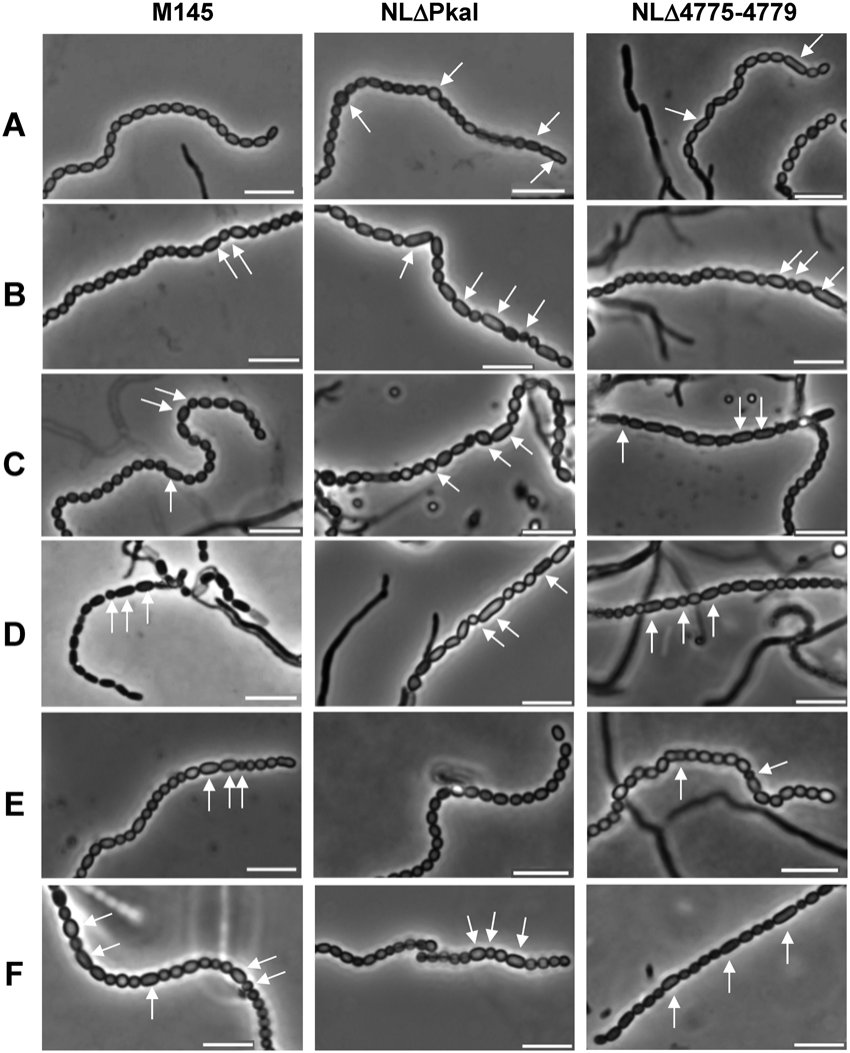
Effect of overbalancing phosphorylation activity on proper sporulation of *S*. *coelicolor*. Phase contrast microscopy of spore chains revealed the presence of aberrant spores (arrows) in eSTPK mutants NLΔPkaI and NLΔ4775–4779 (A). In contrast, spore chains of the parental M145 strain (A) and the complemented mutant NLΔPkaI::pSET-pkaI (E) contain mainly regular ovoid spores. Not only deletion, but also expression of a second copy of any eSTPK gene of cluster *SCO4775-4779* causes a similar sporulation defect (B-F, white arrows) in *S*. *coelicolor* M145, NLΔPkaI, or NLΔ4775–4779. None of the eSTPK genes is able to complement aberrant sporulation of the five-fold mutant NLΔ4775–4779. A, no plasmid integrated; B, :: pSET152-pkaH; C, :: pSET152-SCO4776; D, :: pSET152-pkaD; E, :: pSET152-pkaI; F, :: pSET152-pkaJ. Bar = 5 μm.

The effect of *pkaI* inactivation on spore morphology was studied by seeding cover slides in SM-agar with NLΔPkaI spores and inspecting the slides by phase contrast microscopy after 3–6 days of incubation. Whereas M145 mostly produced very regular spore chains, NLΔPkaI spore chains frequently contained some aberrant spores (Fig 1, arrows) which were enlarged and more spherical. Genetic complementation of NLΔPkaI by integrating an intact copy of *pkaI* together with its native promoter region into the PhiC31 attachment site (NLΔPkaI::pSET-pkaI restored the morphological defect. Both NLΔPkaI phenotypes, the delay in sporulation, as well as the formation of aberrant spores were complemented (Fig 1, S3A and S3B Fig). Whereas integration of pSET-pkaI in the NLΔPkaI chromosome complemented aberrant spore formation, integration of pSET-pkaI into the chromosome of the parental strain M145 caused a similar sporulation defect as the deletion of *pkaI* in NLΔPkaI (Fig 1). Obviously, over-expression of *pkaI* interfered with the control of proper sporulation.

### Overbalancing kinase activity of *S*. *coelicolor* affects integrity of the spore envelope

**Fig 2:**
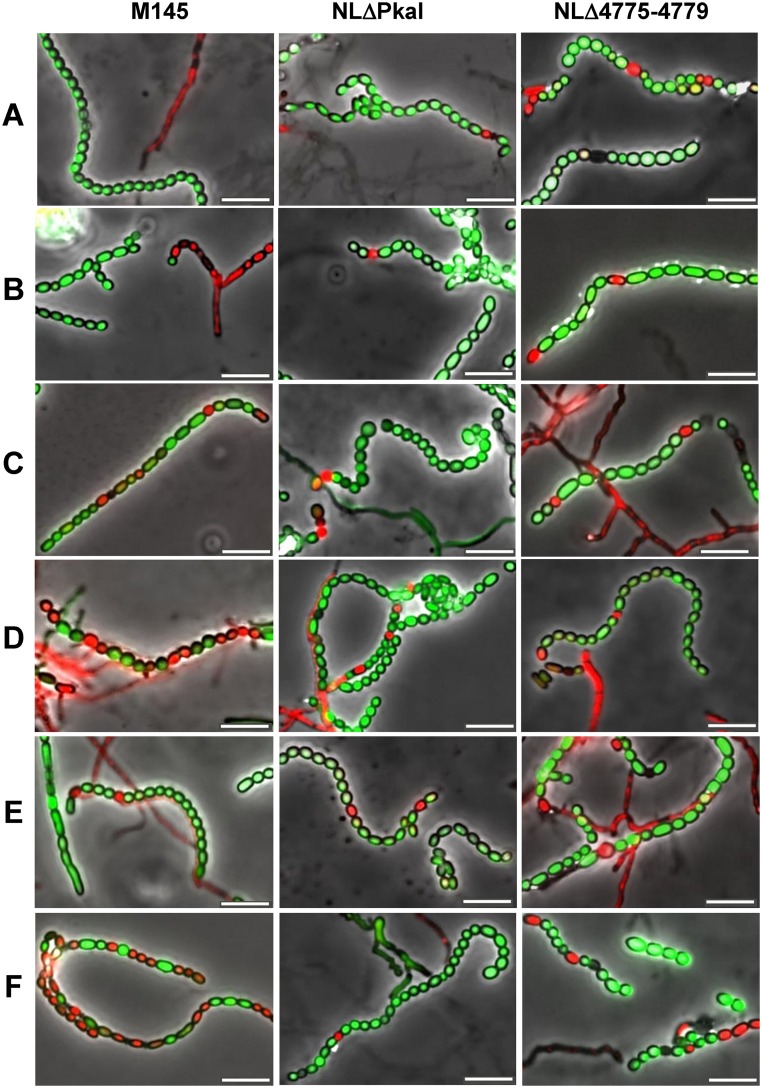
Effect of overbalancing phosphorylation activity on the integrity of spore envelopes. Live-dead staining of spore chains of the eSTPK mutants NLΔPkaI and NLΔ4775–4779 (A) revealed the presence of dead spores (red) or spores without DNA (black). In contrast, spore chains of the parental M145 strain (A) only contained viable spores (green). Expression of a second copy of any eSTPK gene of cluster *SCO4775-4779* (B-F) caused a similar sporulation defect in *S*. *coelicolor* M145, NLΔPkaI, or NLΔ4775–4779. None of the eSTPK genes was able to complement aberrant sporulation of the five-fold mutant NLΔ4775–4779. A, no plasmid integrated; B, :: pSET152-pkaH; C, :: pSET152-SCO4776; D, :: pSET152-pkaD; E, :: pSET152-pkaI; F, :: pSET152-pkaJ. Bar = 5 μm.

**Fig 3:**
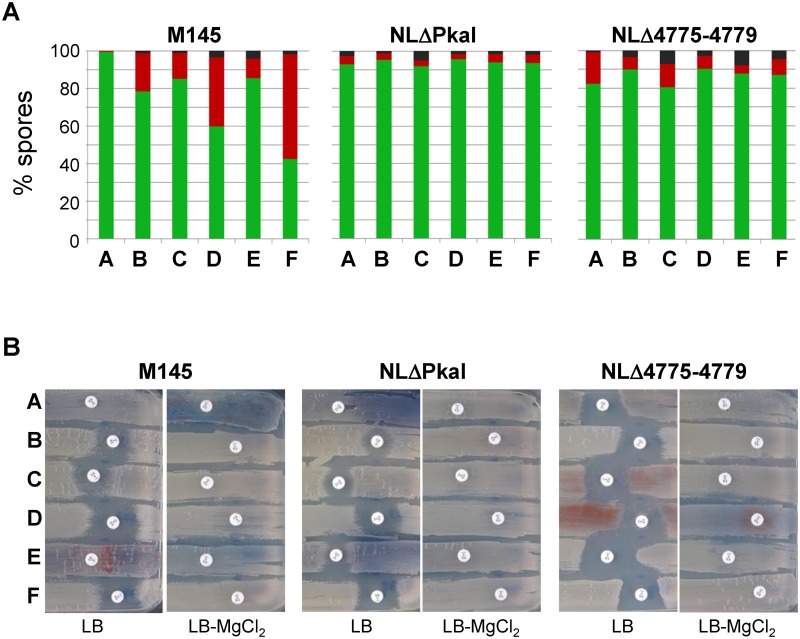
Effect of Ser/Thr kinases on the viability of spores (A) and the resistance of germinating spores to vancomycin (B). Spore chains were stained with the LIVE/DEAD BacLight Bacterial Viability Kit (Molecular Probes) and observed by fluorescence microscopy. Percentage of viable (green), dead (red) and spores without DNA (black) is given for each strain. Spores of the different strains were plated onto LB agar and filter discs containing 5 μg vancomycin were applied. Whereas, M145 and the *pkaI* mutant NLΔPkaI were resistant, NLΔ4775–4779 spores showed vancomycin sensitivity, suggesting an impaired spore wall. Vancomycin sensitivity of M145 or NLΔPkaI was also caused by expressing a second copy of each kinase gene, with the exception of *pkaI*. Supplementation of the agar plates with 3 mM MgCl_2,_ known to rescue mutants impaired in cell wall synthesis restored vancomycin resistance to all strains. A, no plasmid integrated; B, :: pSET152-pkaH; C, :: pSET152-SCO4776; D, :: pSET152-pkaD; E, :: pSET152-pkaI; F, :: pSET152-pkaJ.

Since *pkaI* is located in a genomic region consisting of five consecutive eSTPK genes and since clustering often indicates a functional relationship, we also deleted the complete cluster (S2 Fig). The five-fold eSTPK mutant NLΔ4775–4779 was viable and growth of its substrate mycelium was not affected. Microscopic analyses of NLΔ4775–4779 spore chains revealed beside normally ovoid spores also aberrant rod-shaped spores (Fig 1, arrows). The sporulation defect was even more obvious when testing viability of the spores by SYTO9/propidium iodide staining (Fig 2A, S3 Table). Whereas the wildtype M145 contained almost only (99.28%) viable spores (green), the spore chains of the deletion mutants NLΔPkaI and NLΔ4775–4779, respectively, also included dead (red) spores (4.29% and 16.81%) or sometimes spores even devoid of DNA (black). Moreover, germinating NLΔ4775–4779 spores showed an increased sensitivity towards vancomycin (Fig 3B and S4 Fig), suggesting an impaired spore wall. When vancomycin disks were applied at a later stage, when substrate mycelium has already been formed, NLΔ4775–4779 was as resistant as the NLΔPkaI mutant and M145 (S3C Fig), demonstrating that only the spore wall of NLΔ4775–4779 was sensitive but not the cell wall of the substrate mycelium.

To identify the kinase responsible for the vancomycin sensitivity of the NLΔ4775–4779 spores, we inserted each kinase gene under control of its native promoter region into the ΦC31 attachment site of NLΔ4775–4779, NLΔPkaI and M145. Vancomycin resistance of spores of the five-fold mutant NLΔ4775–4779 as well as the aberrant morphology or the presence of non-viable spores could not be restored by any kinase gene (Figs 1 and 2, S3 Table), suggesting that activities of multiple kinases are required for proper sporulation. Consistent with this observation, introduction of a second copy of any of the kinase genes, with the notable exception of *pkaI*, into M145 or NLΔPkaI caused aberrant spores (Figs 1, 2 and 3) with increased sensitivity to vancomycin (Fig 3B and S4 Fig). This phenotype was most severe, when *pkaJ* was expressed in M145. Here, 59.5% of the spores were non-viable (Fig 3A, S3 Table). Presence of dead spores and vancomycin sensitivity of germinating spores clearly show a defect in the integrity of the spore envelope. Interestingly, vancomycin resistance was restored in all strains, when the spores were plated onto LB supplemented with 3 mM MgCl_2_ (Fig 3B), known to rescue *B*. *subtilis* mutants impaired in cell wall synthesis [38].

These data demonstrate that the elaborated kinase activity of *S*. *coelicolor* is crucial for proper sporulation and any imbalance in the kinase activity by either deletion or over-expression of specific kinases affects sporulation.

### The eSTPK PkaI phosphorylates MreC

**Fig 4:**
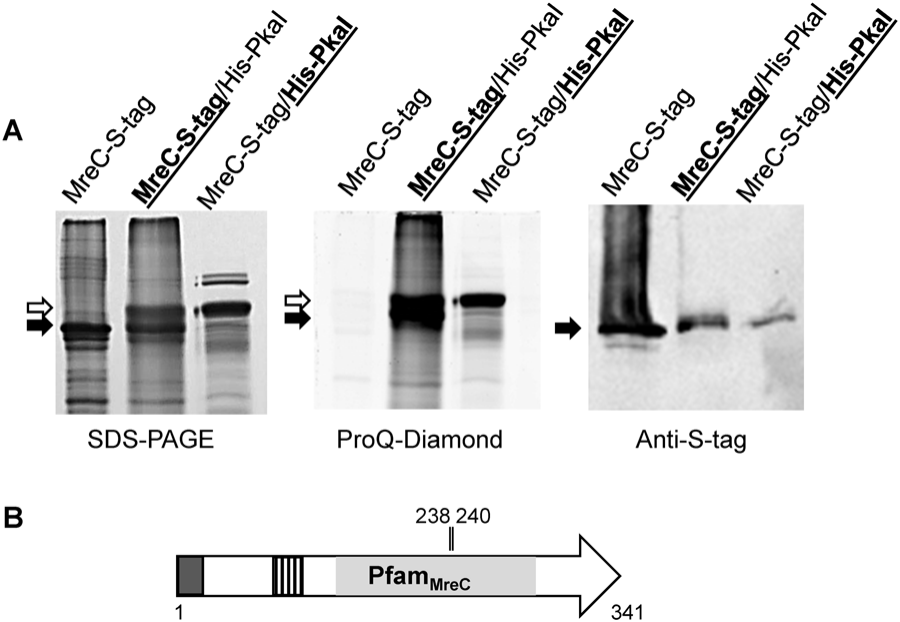
Phosphorylation of MreC by PkaI. *S*. *coelicolor mreC* with a S-tag encoding sequence was expressed in *E*. *coli* in the absence or presence of *pkaI* and purified by affinity chromatography (A). PkaI with an N-terminal His-tag was purified by Ni-NTA chromatography in the presence of *mreC*. Proteins (bold letters and underlining indicate, which protein was purified) were separated on a SDS polyacrylamide gel and stained with Coomassie blue. Purified MreC_S-tag (black arrow) contained considerable amounts of co-purified PkaI (white arrow). Phosphorylated proteins were identified by ProQ Diamond staining. The white arrow indicates autophosphorylated His_PkaI, while the black arrow marks phosphorylated MreC_S-tag. Immunoblotting with Anti-S-tag antibodies confirmed the identity of MreC_S-tag. Domain architecture of *S*. *coelicolor* MreC and positions of the most likely phosphosites (B). Predicted Pfam_MreC domain, signal sequence (dark grey box), coil-coil region (hatched box) and position of phosphorylated T residues, identified by LC-MS/MS, are indicated.

Interaction of the Ser/Thr kinase PkaI with MreC [28] suggested that PkaI phosphorylates MreC, thereby controlling its activity. To demonstrate phosphorylation of MreC by PkaI, we simultaneously expressed both proteins in *E*. *coli* BL21. The respective genes were cloned under control of the P_T7_ promoter in pCDF-Duet1, generating translational fusions either to an N-terminal His-tag encoding sequence (*pkaI*), or a C-terminal S-tag (*mreC*). As controls, MreC_S-tag and His_PkaI were also expressed separately. Following induction of gene expression, MreC_S-tag was purified by S-tag-affinity chromatography and His_PkaI by Ni-NTA chromatography (Fig 4A). After staining the gels with the phosphoserine/phosphothreonine specific dye ProQ Diamond strong bands were visible that corresponded to His_PkaI and MreC_S-tag, respectively, as demonstrated by immunoblotting. Interestingly, the S-tag affinity chromatography always led to two bands (Fig 4A, arrows), both of which were phosphorylated. Mass spectrometry analysis revealed that MreC_S-tag and His_PkaI were present in both bands; the upper band likely represents His_PkaI, which obviously has been co-purified with MreC_S-tag, forming the lower band.

To exclude that phosphorylation of MreC_S-tag occurred accidentally due to the over-expression of His_PkaI, we introduced an additional expression vector (pYT9-Crp) into BL21 carrying pCDF-PkaI-MreC. pYT9-CRP encodes the *S*. *tsukubaensis* Crp protein with a C-terminal Strep-tagII sequence (Crp_strep) under control of the rhamnose inducible P_rham_ promoter (S. Kocadinc, pers. communication). Following induction of gene expression by IPTG and rhamnose, Crp_strep was purified by Streptactin affinity chromatography. ProQ Diamond staining revealed that, in contrast to MreC_S-tag, purified Crp_strep was not phosphorylated (S5 Fig). This demonstrates that phosphorylation of MreC_S-tag by His_PkaI is specific and not caused by the non-physiological expression conditions in the heterologous host.

The phosphosites of MreC were identified using purified MreC_S-tag from *E*. *coli* carrying pCDF-MreC-PkaI. The purified protein was run on a 12.5% SDS polyacrylamide gel, to separate it from contaminating proteins. Following staining of the gel with Coomassie blue, the band corresponding to MreC_S-tag was cut out from the gel and subjected to proteolytic digestion with either trypsin or endoproteinase GluC. Resulting peptide masses were analysed by LC-MS/MS (S4 Table). To discriminate specific phosphorylation sites from artificially phosphorylated residues, the intensities of the phosphorylated peptides were compared to the intensities of the corresponding non-modified peptides; only singly-phosphorylated peptides with at least 10-fold higher intensity than unmodified counterparts were considered. A single MreC-derived peptide was found to be more abundant in the phosphorylated form. The tryptic peptide LVTFGSQADKPFVPGVPVG**p**[**TIT**]RVDPNGGDLTR (bold residues indicate probable phosphorylation sites) comprising MreC_219-251_ was detected 100-fold more abundant compared to the non-phosphorylated one (Table 1). The MreC phosphorylation site (Fig 4B) is localized within the Pfam_MreC domain (MreC_121-273_). Analysis of the fragmentation spectrum did not lead to unambiguous localisation of the phosphorylation to one of the two threonine (**T**_**238**_ **, T**_**240**_) residues.

**Table 1:** Prevalent singly phosphorylated peptides^§^ in MreC, PBP2 and PkaI.

Protein	Peptide	Phospho site	Ratio
MreC*	LVTFGSQADKPFVPGVPVG**p[TIT]**RVDPNGGDLTR	**T**_**238**_ **/T**_**240**_	**0.01**
**PBP2**	GVALADNE**pT**R	**T**_**80**_	**0.004**
	LVVSA**pS**RTDLLK	**S**_**87**_	**0.1**
	SDQVGR**pS**GLER	**S**_**216**_	**0.029**
	AVVSPDGK**pT**VR	**T**_**558**_	**0.007**
**PkaI**	VL**pT**RGPVDAVEAAR	**T**_**113**_	**0.02**
	FGVAQVAGA**p[TT]**LTE	**T**_**173,174**_	**0.000**

^§^ Most probable phosphosites/phosphorylated region, as identified by LC-MS/MS, are highlighted by bold letters. Unlocalized phosphorylation sites are situated in regions marked with brackets. Phosphopeptide ratio represents intensity of non-phosphorylated peptide divided by the intensity of phosphorylated peptide.

* this MreC peptide was also observed in multiple phosphorylated versions, each with a low ratio.

When MreC_S-tag was purified in the absence of PkaI, it was non-phosphorylated (Fig 4A). Also LC-MS analysis detected only non-phosphorylated peptides (data not shown). This demonstrated that MreC was specifically phosphorylated by PkaI and not by an endogenous *E*. *coli* kinase.

### Detection of phosphorylation sites in PkaI

Purified MreC_S-tag also contained considerable amounts of His_PkaI, since S-tag purification obviously did not disrupt the His_PkaI-MreC_S-tag interaction (Fig 4A). Due to their similar size, MreC_S-tag and His_PkaI are not well separated by SDS-PAGE. Therefore, the MreC_S-tag band, which was eluted from the gel to determine the MreC-phosphosites also contained His_PkaI and among the MreC_S-tag peptides also His_PkaI peptides were identified. This further supports the strong interaction of PkaI and MreC, reported by Kleinschnitz et al. [28] and allowed an assignment of the phosphorylation sites not only to MreC but also to PkaI (Table 1 and S4 Table). Two phosphorylated peptides (PkaI_111-124_, VL**pT**RGPVDAVEAAR, PkaI_164-177_, FGVAQVAGA**p[TT]**LTE) were identified for PkaI. Both phosphorylation sites are located in the highly conserved S_TKc kinase domain. PkaI_164-177_ corresponds to the so called activation loop of eSTPKs, involved in determining substrate specificity [39,40] and contains the conserved T residue, which becomes phosphorylated to activate the kinase.

### PkaI phosphorylates also PBP2

**Fig 5:**
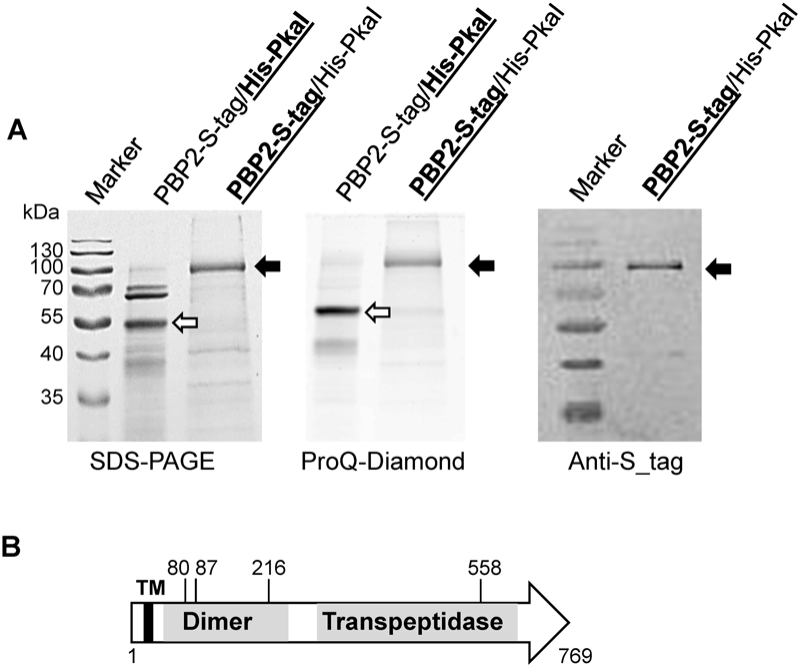
Phosphorylation of PBP2 by PkaI. *S*. *coelicolor pbp2* with a S-tag encoding sequence was co-expressed with *pkaI* in *E*. *coli*. PBP2_S-tag was purified under denaturing conditions by affinity chromatography. PkaI with an N-terminal His-tag was purified by Ni-NTA chromatography under native conditions. Purified His_PkaI and PBP2_S-tag proteins (bold letters and underlining indicate, which protein was purified) were separated on an SDS polyacrylamide gel and stained with Coomassie blue (**A**). Phosphorylated proteins were identified by ProQ Diamond staining. The white arrow indicates auto-phosphorylated His_PkaI, while the black arrow marks phosphorylated PBP2_S-tag. Immunoblotting with Anti-S-tag antibodies confirmed the identity of PBP2_S-tag. Domain architecture of PBP2 and positions of the most likely phosphosites (**B**). Predicted Pfam domains (PBP dimerization, dark grey, PBP transpeptidase, light grey), a transmembrane helix (TM) and the positions of phosphorylated S/T residues, identified by LC-MS/MS, are indicated.

In order to study whether PkaI also phosphorylates other PkaI interaction partners, in addition to MreC, we expressed the monofunctional penicillin binding protein PBP2 together with PkaI. Interestingly, expression of PBP2_S-tag could not be achieved, when *pbp2* alone was inserted in pCDF-Duet1 (S6 Fig). However, in the presence of *his-pkaI*, expression of PBP2_S-tag was obtained in reasonable amounts. Obviously, PkaI had a stabilizing effect on PBP2 expression. Since purification of PBP2_S-tag was not satisfactorily under native conditions, PBP2_S-tag was denatured by the addition of 6 M urea and purified under denaturing conditions (Fig 5A). ProQ diamond staining and immunoblotting with anti-S-tag specific antibodies demonstrated that also purified PBP2-S-tag was phosphorylated. The PBP2-S-tag band was cut from a Coomassie stained polyacrylamide gel, digested with trypsin, and analysed by LC-MS/MS (S4 Table) to identify the phosphorylated peptides (Table 1). PBP2_72-81_ (GVALADNEp**T**R), PBP2_82-93_ (LVVSA**pS**RTDLLK), PBP2_210-220_ (SDQVGR**pS**GLER), and PBP2_550-560_ (AVVSPDGK**pT**VR) were the most prevalent phosphorylated PBP2 peptides (bold letters indicate the phosphosites). Whereas peptide PBP2_550-560_ is located in the transpeptidase domain of PBP2 (Fig 5B), all other PBP2 phosphorylation sites are found in the N-terminal dimerization domain (PBP2_60-250_), suggesting that PBP2 phosphorylation affects its dimerization. LC-MS/MS analyses of the phosphorylated peptides identified T80, S87, S216 and T558 as the residues phosphorylated by PkaI (Table 1).

### PkaI interacts with further proteins involved in differentiation

Previous screening of a *S*. *coelicolor* genomic library for interaction partners of MreC and PBP2 identified PkaI_257-357_ as the interacting domain [28]. To find additional target proteins of PkaI, we screened genomic libraries of *S*. *coelicolor* by bacterial two-hybrid analyses for interaction partners of PkaI. Full length *pkaI*, as well as the fragment encoding the putative interaction domain PkaI_257-357_ were translationally fused to the T18 domain of the *Bordetella pertussis* adenylate cyclase gene *cya*. The resulting plasmids were introduced into the *E*. *coli cya* mutant BTH101. Subsequently, these strains were transformed with genomic libraries of M145 made in the Cya-T25 domain encoding vector pKT25. In total, about 1.7 x 10^6^ colonies were obtained with the enzymatically generated library [28] and about 3 x 10^5^ colonies with a library containing *S*. *coelicolor* DNA fragments generated by nebulization. Transformants encoding interacting fusion proteins were selected on minimal agar supplemented with lactose.

Whereas, not a single colony developed on minimal agar when full length PkaI was fused, numerous colonies grew on minimal agar, when only the PkaI_257-380_ interaction domain was fused to the Cya-T18 domain. Sequence analyses of the pKT25 derivatives confirmed the in frame fusion of most inserts and identified the respective *S*. *coelicolor* proteins which interacted with PkaI (S5 Table).

About 30% of the interacting clones contained a fragment (SCO2097_21-120_) of *SCO2097*. The 135 aa actinomycete signature protein SCO2097 [41] was previously identified as an interaction partner of MreC, MreD, PBP2, Sfr, FtsI and RodZ and was shown to have a role in proper sporulation [28]. Interestingly, several of the PkaI interaction partners, like the putative membrane protein SCO1403 which was isolated five times, or the FtsX-like proteins SCO3110 and SCO3754 have already been identified in the previous screens of SSSC proteins [28]. PkaI also showed self-interaction and interacted with the other eSTPKs PkaA (SCO2974) and PkaD (SCO4777). Moreover, PkaI interacted with several proteins that have a documented role in morphological differentiation, like CrgA (SCO3854), AfsQ1 (SCO4907), BldB (SCO5723) and the FtsH homologue SCO5587. This indicates that PkaI not only phosphorylates specific SSSC proteins but also has a more global role in the regulation of morphological differentiation.

## Discussion

A crucial step in morphological differentiation of mycelial *Streptomyces* is the simultaneous formation of more than 50 septal cross walls in the unbranched aerial hyphae. Sporulation septation and the synthesis of the thickened spore wall involves, besides SsgA-like proteins and FtsZ [42–44], the SSSC, a multi-protein complex for spore envelope synthesis that highly resembles the elongasome of rod-shaped bacteria [28]. But whereas, rod-shaped bacteria build only a single septum during cell division, *Streptomyces* faces the problem, how to build dozens of septal cross walls at the same time [42]. For this process the membrane- and PG-synthesizing machineries have to be provided in sufficient quantities and positioned properly. Moreover, the activities of the complexes have to be controlled to prevent aberrant sporulation by sporadic formation of single cross walls in a non-coordinated manner.

In this study we are describing a route, how the control of sporulation septation and the synthesis of the spore wall could occur by protein phosphorylation.

The crucial role of the eSTPKs PkaH, SCO4776, PkaD, PkaI and PkaJ in sporulation was apparent in gene inactivation and overexpression studies. Deletion of *pkaI* or all five eSTPK genes, as well as expression of a second copy of any of these kinases produced aberrant spore chains, containing irregular-shaped spores. SYTO9/ propidium iodide staining revealed the presence of spores, which were considerably affected in the integrity of the spore envelope. Moreover the germinating spores of the five-fold deletion mutant and the over-expressing strains were highly sensitive to vancomycin, whereas the spores of the wildtype and the *pkaI* mutant NLΔPkaI were resistant, due to the presence of the *S*. *coelicolor vanHAX* resistance determinants. Although the rationale for the increased sensitivity of germinating spores of the SSSC mutants to different kinds of stress, including cell wall damaging agents, like lysozyme or vancomycin [27, 28, 29] is not understood, it suggests an impaired spore wall. Since the transpeptidase PBP2 is a key protein of the SSSC, the SSSC mutants are probably affected in the crosslinking of the spore wall. Reduced crosslinking of the PG-layer would correlate with an increase in the vancomycin binding sites (D-ala-D-ala). The increased amount of bound vancomycin then probably interferes with spore germination.

Phosphorylation of proteins as a regulatory mechanism to control their activity is well documented in many bacteria and various proteins of the divisome were shown to be phosphorylated [45]. For example, the mycobacterial kinase PknA was shown to affect septum formation by inhibiting the GTP-dependent polymerization of FtsZ [46]. PknA also phosphorylates the PG-synthesis ligase MurD [47] and the mycobacterial DivIVA homolog Wag31, crucial for polar growth [48]. Activity of DivIVA of *S*. *coelicolor*, which directs growth by apical tip extension and hyphal branching, is controlled by the protein kinase AfsK [22].

Here we show for the first time that MreC and PBP2, two important proteins in spore wall synthesis of *S*. *coelicolor* are specifically phosphorylated. In contrast, MreC and PBP2 of rod-shaped bacteria have not been reported to be phosphorylated. Neither recently published phosphoproteome studies [49,50], nor inspection of phospho-proteome databases (PhosSite and Phosida) of *B*. *subtilis* and *E*. coli indicated phosphorylation of MreC or any other Mre protein of *E*. *coli* or *B*. *subtilis*.

*In vivo* phosphorylation of MreC and PBP2 by PkaI was demonstrated in *E*. *coli*. Authenticity of MreC_S-tag phosphorylation is confirmed by the control experiments, which showed non-phosphorylation of Strep-tag_Crp and lack of MreC phosphorylation in the absence of *pkaI*. Direct detection of phosphorylated MreC or PBP2 in *S*. *coelicolor* was not possible, since over-expression of each of these proteins is toxic (unpublished results), preventing the production of sufficient amounts for the detection of phosphorylated peptides. In published datasets of *S*. *coelicolor* [51,52] MreC or PBP2 are not included, indicative of their low expression level. Expression of PBP2 in *E*. *coli* in this study was also problematic and could only be achieved in the presence of PkaI (S6 Fig), but not in the absence of *pkaI*. The stabilizing effect of *pkaI* on PBP2 could be due to toxicity of *pbp2* expression, probably caused by interference of PBP2 with the *E*. *coli* PG-synthesizing machinery. Since three of the four PBP2 phosphosites are located within the dimerization domain, it is tempting to speculate that phosphorylation of PBP2 prevents its dimerization, thus inactivating PBP2 and allowing its expression in *E*. *coli*.

On the first view, phosphorylation of extracellular MreC or PBP2 domains by the cytosolic kinase domain of PkaI seems to be implausible. But if the purpose of phosphorylation is to keep the synthesized proteins inactive, the topology of the phosphosites is of minor relevance. Moreover, the phosphosites of the penicillin binding protein PBPA of *Mycobacterium tuberculosis*, phosphorylated by the eSTPK PknB are also in the (extracellular) transpeptidase domain [31]. Here it was shown that phosphorylation affected positioning of PBPA at the septum, thereby regulating septal peptidoglycan biosynthesis.

Expression of PkaI (and PkaD, PkaJ) was found to be highest in the early growth phase and down regulated in later stages of differentiation. This expression profile is in agreement with microarray data of *S*. *coelicolor* [53], which confirm our semi-quantitative RT-PCR data. The only discrepancy concerns *pkaH*, which was highly expressed in the Yagüe-study [53], while its expression was hardly detectable under our growth conditions. However, the *pkaI* expression profile differs from the expression of *mreC* and *pbp2*, which are induced during morphological differentiation. The different expression profiles of the kinase PkaI and their substrates MreC and PBP2 do not seem to be consistent with the observed phosphorylation of MreC and PBP2 by PkaI. However, this apparent contradiction might be the key for understanding how sporulation septation is controlled by phosphorylation of SSSC proteins. As indicated by the inability to produce PBP2 in the absence of *pkaI*, phosphorylation of PBP2 might interfere with its activities, probably by affecting subcellular positioning, dimerization, or interaction with other proteins to assemble a functional SSSC. As consequence, the PkaI-mediated phosphorylation would prevent the premature formation of sporadic SSSC complexes unless outgrowth of aerial hyphae is finished and enough SSSC proteins have been synthesized to allow coordinated assembly of more than 50 SSSC complexes. Also, any imbalance in kinase activity, caused by over-expression of single kinase genes interferes with proper sporulation by affecting the coordinated assembly of the SSSC. Control of morphological differentiation most probably also includes one of the 55 *S*. *coelicolor* phosphatases [32], able to activate phosphorylated SSSC proteins by dephosphorylation.

The role of PkaI in inhibition of sporadic SSSC complexes is strengthened by the observed interaction of PkaI with CrgA, a septation inhibitor protein that coordinates growth and cell division in aerial hyphae [54]. Interaction of PkaI with other sporulation proteins, like SCO2097, AfsQ, FtsH, FtsX-like proteins, and BldB suggests a more global role of PkaI in differentiation, besides controlling the SSSC. Overexpression of *bldB* blocked sporulation in aerial hyphae and evidence for the interaction of BldB with an unknown cellular constituent involved in differentiation was reported [55].

The proposed prominent role of PkaI in differentiation of *S*. *coelicolor* is in contrast to the quite mild phenotype of *pkaI* inactivation. However, single eSTPK mutants often only have a mild phenotype [56,57], probably due to the well documented cross reactivity of eSTPK proteins [45]. In *S*. *coelicolor*, other eSTPKs probably can substitute the missing PkaI activity. This is supported by the more severe phenotype of the five-fold mutant NLΔ4775–4779 and by our protein-protein interaction data which revealed interaction of PkaI with the other eSTPKs PkaA and PkaD. Moreover, deletion mutants of various SSSC proteins do not completely block sporulation but cause only spores with impaired envelopes [21,27,28], suggesting that the defects can be suppressed by redundant enzymatic activities. In agreement with this, at least four PBPs have been identified in the SSSC [28,29].

Clearly, further experimental work is required to support the proposed differentiation model. The phosphorylation of SSSC proteins should be confirmed in *S*. *coelicolor* to reflect their native environment and the biological effect of SSSC phosphorylation has to be demonstrated, e.g. by introducing phosphomimetic mutations. Unfortunately, no enzymatic assays are available for any SSSC protein to directly quantify the effects of protein phosphorylation on the activity of the respective protein.

## Material and Methods

### Bacterial strains and media

Cultivation of strains and procedures for DNA manipulation were performed as previously described [58,59]. Proteins were purified from BL21 (DE3) (Invitrogen). Plasmids and oligonucleotides are listed in S1 and S2 Tables.

### RNA isolation and cDNA synthesis

M145 was grown on cellophane disks placed on SM agar plates. Plates were incubated for either 24, 48, 72 or 96 hours at 30°C. The mycelium was harvested and lysed in Kirby-Mix [59] with a Precellys homogenizer (Peqlab; 5 times program: 6500rpm 2 x 20sec). RNA isolation was carried out as described by Kieser et al. [59] with minor modifications. RevertAid RT Kit (Thermo) was used to synthesize cDNA from isolated RNA according to manufacturer´s protocol.

### Construction of mutants

To delete *pkaI (SCO4778)*, a 1.6 kb upstream fragment (primer pair Up4778fwE/ Up4778revB) including the start codon of *sco4778* and a 1.6 kb downstream fragment (primer pair lo4778fwB/ lo4778revH) including the *sco4778* stop codon was amplified by PCR and cloned into pKT18, yielding pKO4778. After transformation of M145 and integration of the deletion vector pKO4778 by a single crossover (kanamycin-resistant), a Δ*pkaI* mutant was isolated by selecting for the second crossover (kanamycin-sensitive). Correct gene replacement was confirmed by PCR-analyses and Southern-blotting. The hybridization probe was amplified with primers Up4778fwE/ Up4778revB.

To delete all five serine/threonine kinase genes, a 1.6 kb upstream fragment (primer pair Up4775Ef/Up2775Br) including the start codon of *SCO4775* and a 1.6 kb downstream fragment (primer pair Lo4779Bf/Lo4779Hr) including the *SCO4779* stop codon were amplified and cloned into pK18. Subsequently the knockout cassette was cut out with EcoRI/HindIII and inserted in pGus21, yielding pGusKO4775-4779.

pGusKO4775-4779 was introduced into *S*. *coelicolor* M145 by intergeneric conjugation [60] and apramycin resistant transconjugants were selected that carried pGusKO4775-4779 integrated via a single crossover (M145::pGusKO4775-4779). To screen for the second cross over, resulting in *SCO4775-SCO4779* deletion, M145::pGusKO4775-4779 was plated onto soja mannit (SM) agar without antibiotic and incubated for five days. Spores were harvested and appropriate dilutions were plated onto LB agar to obtain single colonies. After two days incubation at 30°C, plates were overlayed with 1 ml H_2_O containing 2.5 mg X-gluc. Colonies that still carried pGusKO4775-4779 were surrounded by a blue halo due to the 4-Cl-3-Br-indigo production by the β-glucuronidase (GusA). Colonies that had lost pGusKO4775-4779 by the second cross over were identified by the lack of the blue halo. Deletion of *SCO4775-SCO4779* was confirmed by PCR analyses using primers Intern4778f/Intern4778r, Up4778K416/Lp4778K2240, and c4775-79fwEX/c4775-79revEX.

### Spore production assay

Approximately 3x10^7^ spores of the respective strain were plated on soya mannit (SM) agar. The plates were incubated at 30°C for 3 and 7 days, respectively, before harvesting the newly formed spores. Dilutions of the spore samples were plated in duplicate to determine the spore titer. 6 biological replicates were made from M145 and NLΔPkaI; 8 replicates from NLΔ4775–4779 and NLΔPkaI::pSET-pkaI.

### Heterologous co-expression

*pkaI* (*SCO4778*), *mreC* (*SCO2610*) and *pbp2* (*SCO2608*) were amplified using the primers listed in S2 Table, digested with the respective enzymes, and cloned into the vector pCDFDuet1 (Merck/Novagen). The resulting plasmids (S1 Table) were transferred into *E*. *coli* BL21 (DE3) for protein production. Expression of the proteins was performed as described by [61]. Briefly, cells were grown in LB medium containing 100μg spectinomycin/ml at 37°C until an OD600 of 0.6–0.8. Then expression was induced with IPTG at a final concentration of 0.5mM for 3h at 37°C. Purification of S-tagged MreC protein was carried out according to the manufacturers protocol (Novagen) with minor modifications. His-tagged PkaI was purified via Ni-NTA sepharose gravity flow columns using the manufacturers protocol (IBA). As lysis, washing and elution buffer we used 20mM Tris/HCl buffer, pH 7.5 with 150mM NaCl and 0.1% Triton X100. PBP2_S-tag was purified under denaturating conditions by adding urea to the buffer to a final concentration of 6M.

Expression of the Strep-tagII-Crp protein of *Streptomyces tsukubaensis* (*STSU_15619* gene), used as a negative control, was induced with rhamnose at a final concentration of 0.02%. Strep-tagII-CRP was purified using an Äkta purifier (GE Healthcare) with a StrepTrap HP columns following the manufacturers protocol (GE Healthcare). Identity of the purified proteins was confirmed by immunoblotting. Following protein transfer (45 minutes at 400mA) to nitrocellulose membranes, the membranes were incubated, washed and developed according to the protocol for the respective antibody (Anti-S-tag-HRP, Novagen; Anti-Strep-tag-HRP, Biorad; Anti-His6 HRP, Bethyl Laboratories, Inc)

### Phospho-serine and -threonine detection

Purified proteins were run on 12.5% SDS-polyacrylamide gels. To detect phosphorylation on serine or threonine residues the gels were stained with ProQ Diamond (Molecular Probes) according to the manufacturers recommendations.

### Microscopy

About 10^6^ spores were plated onto MS agar and sterile coverslips were inserted in a certain angle of 45°. After five to seven days of incubation at 30°C the coverslips were removed and mounted on slides coated with 1% agarose in PBS.

To detect dead spores, SYTO9 and propidium iodide stains of the LIVE/DEAD BacLight Bacterial Viability Kit (Molecular Probes) were used. The staining solution was prepared by mixing 1.5μl of component A and B in 1ml of water. Spores were incubated on the agar plate with 20μl of staining solution for 15 minutes, then coverslips were removed and mounted on slides coated with 1% agarose in PBS. Images were taken with an Olympus System Microscope BX60 equipped with a F-view II camera (Olympus), using TxRed and eGFP filtersets for detection of the fluorescent markers. Fiji version v.149b was used for image processing and the Cell Counter Plugin for spore counting. Live-dead percentage was calculated from the analyses of ~ 700–1800 spores from each strain (S3 Table).

### Nano LC-MS/MS analysis

Samples were loaded on a NuPAGE Bis-Tris 4%–12% gradient gel (Invitrogen) and the Coomassie stained bands were pooled and in gel digested with trypsin and GluC, respectively, as described elsewhere [62]. LC-MS analyses of the peptides were done on an EasyLC nano-HPLC (Proxeon Biosystems) coupled to an LTQ Orbitrap Elite mass spectrometer (Thermo Scientific) as described elsewhere [63]. MS data were processed using the software suite MaxQuant, version 1.2.2.9 [64] and searched using Andromeda search engine [65] against a target-decoy *E*. *coli* database containing 4,311 forward protein sequences, the sequences of the tagged and overexpressed proteins and 248 frequently observed protein contaminants. Trypsin or GluC, were set as proteases in which two missed cleavage sites were allowed. Carbamidomethylation of cysteine was set as fixed modification; N-terminal acetylation, methionine oxidation and serine/threonine/tyrosine phosphorylation were set as variable modifications. Initial precursor mass tolerance was set to 6 parts per million (ppm) at the precursor ion and 20 ppm at the fragment ion level. False discovery rates were set to 1% at peptide, phosphorylation site, and protein group level.

### Bacterial two-hybrid interaction assays

*pkaI* and a *pkaI* fragment encoding only the putative PkaI interaction domain (PkaI_257-380_) were amplified with primers (listed in S2 Table) containing XbaI and KpnI sites, respectively. Subsequently, PCR fragments were cloned with XbaI/KpnI into plasmid pUT18c to generate translational fusions with the catalytic T18 domain of the *B*. *pertussis* adenylate cyclase [66] and introduced into the *E*. *coli cya* mutant BTH101. These strains were electroporated with the *S*. *coelicolor* genomic libraries, either constructed by cloning 250 bp—2000 bp DNA fragments obtained by a partial BfuCI digest [28], or 250–1000 bp fragments generated by shearing the chromosomal DNA in a nebulizer device (Invitrogen) (Tesfazgi & Muth, unpublished), into pKT25. Transformation mixes were plated onto M63 minimal agar containing kanamycin and ampicillin to select for the presence of pUT18c-4778 and a library derived pKT25 derivative. The ability of co-transformants to use lactose resulting in growth on minimal agar is based on a functional adenylate cyclase due to the interaction of the fusion proteins. From growing colonies plasmid DNA was isolated and used in retransformation experiments of BTH101 (pUT18c-4778) to confirm the interaction. From still positive clones, pKT25 was isolated and sequenced to identify the encoded protein and to confirm in frame fusion to the T25 domain.

## Supporting Information

S1 Fig Organisation and expression profile of the *SCO4775-4779* cluster encoding five eSTPKs involved in morphological differentiation. Schematic drawing of the *SCO4775-SCO4779* genes in *S*. *coelicolor* M145 (**A**). Sizes of intergenic regions (grey triangles) are given. Analyses of the operon structure (**B**) and expression profile (**C**) during the *S*. *coelicolor* life cycle. Cultures of *S*. *coelicolor* M145 were grown on cellophane discs on SM agar for different time periods representing distinct stages of the life cycle (indicated in **C**). Following RNA isolation and cDNA synthesis, the intergenic regions of *SCO4775-SCO4779* were amplified by PCR (**B**) using primers, indicated in the schematic drawing. The absence of a PCR product indicates that each gene is transcribed separately. 1. 24h, 2: 48h, 3: 96h, 4: 168h, D: M145 genomic DNA, M: 1kb ladder, Fermentas. Using gene specific primers (listed in S2 Table), the amounts of transcripts during the life cycle were compared by PCR. Amount of 16 S RNA served as an internal control.
(EPS)

S2 Fig Confirmation of NLΔPkaI and NLΔ4775–4779 genotypes by Southern blotting and PCR analyses. Total DNA of M145 and two clones of NLΔPkaI were digested with SmaI and hybridized with a probe corresponding to the upstream region of *pkaI*. **A**. Schematic drawing. The black bar indicates the probe. Only relevant SmaI sites (S) are given. The grey dotted line indicates the hybridysing SmaI fragment. **B.** Southern blot. 1: pKO-4778, 2: M145 DNA, 3: NLΔPkaI-clone 1, 4: NL-PkaI-clone 2, 5: M145 DNA, M: DigVII Standard, Roche. The sizes of the hybridysing bands (arrows) are given.
Total DNA of M145 (1), NLΔPkaI (2) and NLΔ4775_4779 (3) were used to amplify an internal *pkaI* fragment using primers Intern4778f/Intern4778r (Af/Ar) (**C**), the complete *pkaI* with 428 bp upstream and 154 bp downstream region (primers Up4778K416/Lp4778K2240; Bf/Br) (**D**), or the eSTPK gene cluster comprising *SCO4775-SCO4779* with primers c4775-79fw/c4775-79rev (Cf/Cr) **(E)**. M: 1 kb ladder, Fermentas, 1: M145-DNA, 2: NLΔPkaI-DNA, 3: NLΔ4775-4779-DNA. Primer binding sites are indicated (arrows) in the schematic drawing (**A**).
(EPS)

S3 Fig Sporulation ability of the eSTPK mutants NLΔPkaI and NLΔ4775–4779. Spores of M145 and the mutants NLΔPkaI and NLΔ4775–4779 were plated on SM-agar. After 2, 3 and 7 days of incubation, photos were taken (**A**). While NLΔPkaI is delayed in sporulation, differentiation of NLΔ4775–4779 does not visibly differ from that of M145. Genetic complementation of NLΔPkaI restored sporulation proficiency. 1: *S*. *coelicolor* M145, 2: NLΔPkaI, 3: NLΔPkaI::pSET-pkaI, 4: NLΔ4775–4779, 5: NLΔ4775–4779::pSET-pkaI. The delay in sporulation is consistent with the amount of spores that could be isolated after 3 and 7 days of incubation, respectively (**B**). Whereas resistance of germinating spores to lysozyme and vancomycin was not affected in NLΔPkaI, NLΔ4775–4779 spores showed an increased sensitivity (**D**), suggesting an impaired spore wall. In contrast, vancomycin resistance of vegetative mycelium of NLΔ4775–4779 was indistinguishable from that of M145 (right panel).
(EPS)

S4 Fig Effects of unbalancing phosphorylation activity on vancomycin and lysozyme resistance. About 10^5^ spores were streaked on LB agar and serial dilutions of vancomycin and lysozyme spotted. Plates were incubated for three days at 30°C. Whereas resistance of germinating spores to lysozyme was not dramatically affected, spores of the five-fold mutant NLΔ4775–4779, as well as the kinase overexpressing strains (with the exception of *pkaI*) were highly sensitive to vancomycin.
(EPS)

S5 Fig Controls for specificity of PkaI phosphorylation. *pkaI* and *mreC* of *S*. *coelicolor* were cloned into pCDFDUET1 under control of the T7 promoter. *crp* of *S*. *tsukubaensis* was inserted into pYT9 under control of the rhamnose inducible P_rham_ promoter (**A**). *E*. *coli* BL21 was co-transformed with both plasmids. Following induction of gene expression by 1 mM IPTG and 0.2% rhamnose, His-PkaI, MreC-S-tag, and CRP-Strep-tag were purified by affinity chromatography. Purified proteins were loaded to an SDS polyacrylamide gel (**B**) and phosphorylated proteins were identified by ProQ Diamond (Molecular Probes) staining (**C**). The black arrow mark phosphorylated His-PkaI and MreC-S-tag proteins, while the white arrow indicates Crp which was not phosphorylated. PkaI specifically phosphorylates MreC but does not phosphorylate Crp of *S*. *tsukubaensis*. 1: PkaI purified via His-tag, 2: MreC purified via S-tag, 3: Crp purified via Strep-tag from BL21(pCDF-pkaI/pYT9-Crp), 4: Crp purified via Strep-tag from BL21 (pCDF-pkaI-MreC/pYT9-Crp).
(EPS)

S6 Fig Lack of PBP2-S-tag expression in the absence of PkaI. Pellet and supernatant fractions of *E*. *coli* BL21 carrying either pCDF-PkaI or pCDF-PkaI-PBP2 were analysed for the presence of PBP2-S-tag (arrow) by immunoblotting with Anti-S-tag specific antibodies, after induction with 1 mM IPTG for 0.5 and 2 h, respectively.
(EPS)

S1 Table Plasmids. (DOCX)

S2 Table Primer. (DOCX)

S3 Table Live-dead. (XLSX)

S4 Table Phosphorylated peptides. (XLSX)

S5 Table S. coelicolor interaction partners of the eSTPK PkaI. (DOCX)

## References

[1] Glauner B, Holtje JV, Schwarz U (1988) The composition of the murein of *Escherichia coli* . J Biol Chem 263: 10088–10095. 3292521

[2] Schleifer KH, Kandler O (1972) Peptidoglycan types of bacterial cell walls and their taxonomic implications. Bacteriol Rev 36: 407–477. 4568761 10.1128/br.36.4.407-477.1972 PMC408328

[3] Maniloff J (1983) Evolution of wall-less prokaryotes. Annu Rev Microbiol 37: 477–499. 6195959 10.1146/annurev.mi.37.100183.002401

[4] Brown S, Santa Maria JP Jr., Walker S (2013) Wall teichoic acids of gram-positive bacteria. Annu Rev Microbiol 67: 313–336. 10.1146/annurev-micro-092412-155620 24024634 PMC3883102

[5] Chin T, Burger MM, Glaser L (1966) Synthesis of teichoic acids. VI. The formation of multiple wall polymers in *Bacillus subtilis* W-23. Arch Biochem Biophys 116: 358–367. 4960203 10.1016/0003-9861(66)90042-7

[6] Vollmer W (2008) Structural variation in the glycan strands of bacterial peptidoglycan. FEMS Microbiol Rev 32: 287–306. 18070068 10.1111/j.1574-6976.2007.00088.x

[7] Cabeen MT, Charbon G, Vollmer W, Born P, Ausmees N, Weibel DB et al (2009) Bacterial cell curvature through mechanical control of cell growth. EMBO J 28: 1208–1219. 10.1038/emboj.2009.61 19279668 PMC2683044

[8] Pinho MG, Kjos M, Veening JW (2013) How to get (a)round: mechanisms controlling growth and division of coccoid bacteria. Nat Rev Microbiol 11: 601–614. 10.1038/nrmicro3088 23949602

[9] Carballido-Lopez R, Errington J (2003) The bacterial cytoskeleton: in vivo dynamics of the actin-like protein Mbl of *Bacillus subtilis* . Dev Cell 4: 19–28. 12530960 10.1016/s1534-5807(02)00403-3

[10] Daniel RA, Errington J (2003) Control of cell morphogenesis in bacteria: two distinct ways to make a rod-shaped cell. Cell 113: 767–776. 12809607 10.1016/s0092-8674(03)00421-5

[11] van den Ent F, Amos LA, Lowe J (2001) Prokaryotic origin of the actin cytoskeleton. Nature 413: 39–44. 11544518 10.1038/35092500

[12] Carballido-Lopez R, Errington J (2003) A dynamic bacterial cytoskeleton. Trends Cell Biol 13: 577–583. 14573351 10.1016/j.tcb.2003.09.005

[13] Defeu Soufo HJ, Graumann PL (2004) Dynamic movement of actin-like proteins within bacterial cells. EMBO Rep 5: 789–794. 15272301 10.1038/sj.embor.7400209 PMC1299120

[14] Kim SY, Gitai Z, Kinkhabwala A, Shapiro L, Moerner WE (2006) Single molecules of the bacterial actin MreB undergo directed treadmilling motion in *Caulobacter crescentus* . Proc Natl Acad Sci U S A 103: 10929–10934. 16829583 10.1073/pnas.0604503103 PMC1544151

[15] Dominguez-Escobar J, Chastanet A, Crevenna AH, Fromion V, Wedlich-Soldner R, Carballido-Lopez R (2011) Processive movement of MreB-associated cell wall biosynthetic complexes in bacteria. Science 333: 225–228. 10.1126/science.1203466 21636744

[16] Garner EC, Bernard R, Wang W, Zhuang X, Rudner DZ, Mitchison T (2011) Coupled, circumferential motions of the cell wall synthesis machinery and MreB filaments in *B*. *subtilis* . Science 333: 222–225. 10.1126/science.1203285 21636745 PMC3235694

[17] Kawai Y, Asai K, Errington J (2009) Partial functional redundancy of MreB isoforms, MreB, Mbl and MreBH, in cell morphogenesis of *Bacillus subtilis* . Mol Microbiol 73: 719–731. 10.1111/j.1365-2958.2009.06805.x 19659933

[18] Bean GJ, Flickinger ST, Westler WM, McCully ME, Sept D, Weibel DB et al (2009) A22 disrupts the bacterial actin cytoskeleton by directly binding and inducing a low-affinity state in MreB. Biochemistry 48: 4852–4857. 10.1021/bi900014d 19382805 PMC3951351

[19] Kruse T, Bork-Jensen J, Gerdes K (2005) The morphogenetic MreBCD proteins of Escherichia coli form an essential membrane-bound complex. Mol Microbiol 55: 78–89. 15612918 10.1111/j.1365-2958.2004.04367.x

[20] Formstone A, Carballido-Lopez R, Noirot P, Errington J, Scheffers DJ (2008) Localization and interactions of teichoic acid synthetic enzymes in *Bacillus subtilis* . J Bacteriol 190: 1812–1821. 18156271 10.1128/JB.01394-07 PMC2258661

[21] Mazza P, Noens EE, Schirner K, Grantcharova N, Mommaas AM, Koerten HK, et al (2006) MreB of *Streptomyces coelicolor* is not essential for vegetative growth but is required for the integrity of aerial hyphae and spores. Mol Microbiol 60: 838–852. 16677297 10.1111/j.1365-2958.2006.05134.x

[22] Hempel AM, Wang SB, Letek M, Gil JA, Flardh K (2008) Assemblies of DivIVA mark sites for hyphal branching and can establish new zones of cell wall growth in *Streptomyces coelicolor* . J Bacteriol 190: 7579–7583. 10.1128/JB.00839-08 18805980 PMC2576665

[23] Flardh K (2003) Essential role of DivIVA in polar growth and morphogenesis in *Streptomyces coelicolor* A3(2). Mol Microbiol 49: 1523–1536. 12950918 10.1046/j.1365-2958.2003.03660.x

[24] Fuchino K, Bagchi S, Cantlay S, Sandblad L, Wu D, Bergman J, et al (2013) Dynamic gradients of an intermediate filament-like cytoskeleton are recruited by a polarity landmark during apical growth. Proc Natl Acad Sci U S A 110: E1889–1897. 10.1073/pnas.1305358110 23641002 PMC3666699

[25] Holmes NA, Walshaw J, Leggett RM, Thibessard A, Dalton KA, Gillespie MD, et al (2013) Coiled-coil protein Scy is a key component of a multiprotein assembly controlling polarized growth in *Streptomyces* . Proc Natl Acad Sci U S A 110: E397–406. 10.1073/pnas.1210657110 23297235 PMC3562780

[26] Burger A, Sichler K, Kelemen G, Buttner M, Wohlleben W (2000) Identification and characterization of the mre gene region of *Streptomyces coelicolor* A3(2). Mol Gen Genet 263: 1053–1060. 10954092 10.1007/s004380050034

[27] Heichlinger A, Ammelburg M, Kleinschnitz EM, Latus A, Maldener I, Flardh K, et al (2011) The MreB-like protein Mbl of *Streptomyces coelicolor* A3(2) depends on MreB for proper localization and contributes to spore wall synthesis. J Bacteriol 193: 1533–1542. 10.1128/JB.01100-10 21257777 PMC3067644

[28] Kleinschnitz EM, Heichlinger A, Schirner K, Winkler J, Latus A, Maldener I, et al (2011) Proteins encoded by the *mre* gene cluster in *Streptomyces coelicolor* A3(2) cooperate in spore wall synthesis. Mol Microbiol 79: 1367–1379. 10.1111/j.1365-2958.2010.07529.x 21244527

[29] Sigle S, Ladwig N, Wohlleben W, Muth G (2014) Synthesis of the spore envelope in the developmental life cycle of *Streptomyces coelicolor* . Int J Med Microbiol. 305:183–189 10.1016/j.ijmm.2014.12.014 25595023

[30] Petrickova K, Petricek M (2003) Eukaryotic-type protein kinases in *Streptomyces coelicolor*: variations on a common theme. Microbiology 149: 1609–1621. 12855714 10.1099/mic.0.26275-0

[31] Urabe H, Aoyagi N, Ogawara H, Motojima K (2008) Expression and characterization of the *Streptomyces coelicolor* serine/threonine protein kinase PkaD. Biosci Biotechnol Biochem 72: 778–785. 18323658 10.1271/bbb.70658

[32] Shi L, Zhang W (2004) Comparative analysis of eukaryotic-type protein phosphatases in two streptomycete genomes. Microbiology 150: 2247–2256. 15256567 10.1099/mic.0.27057-0

[33] Cole JL (2007) Activation of PKR: an open and shut case? Trends Biochem Sci 32: 57–62. 17196820 10.1016/j.tibs.2006.12.003 PMC2703476

[34] Mir M, Asong J, Li X, Cardot J, Boons GJ, Husson RN (2011) The extracytoplasmic domain of the *Mycobacterium tuberculosis* Ser/Thr kinase PknB binds specific muropeptides and is required for PknB localization. PLoS Pathog 7: e1002182 10.1371/journal.ppat.1002182 21829358 PMC3145798

[35] Fleurie A, Manuse S, Zhao C, Campo N, Cluzel C, Lavergne JP, et al (2014) Interplay of the serine/threonine-kinase StkP and the paralogs DivIVA and GpsB in pneumococcal cell elongation and division. PLoS Genet 10: e1004275 10.1371/journal.pgen.1004275 24722178 PMC3983041

[36] Fiuza M, Canova MJ, Patin D, Letek M, Zanella-Cleon I, Becchi M., et al (2008) The MurC ligase essential for peptidoglycan biosynthesis is regulated by the serine/threonine protein kinase PknA in *Corynebacterium glutamicum* . J Biol Chem 283: 36553–36563. 10.1074/jbc.M807175200 18974047 PMC2662310

[37] Jolly L, Ferrari P, Blanot D, Van Heijenoort J, Fassy F, Mengin-Lecreulx D, (1999) Reaction mechanism of phosphoglucosamine mutase from *Escherichia coli* . Eur J Biochem 262: 202–210. 10231382 10.1046/j.1432-1327.1999.00373.x

[38] Formstone A, Errington J (2005) A magnesium-dependent mreB null mutant: implications for the role of *mreB* in *Bacillus subtilis* . Mol Microbiol 55: 1646–1657. 15752190 10.1111/j.1365-2958.2005.04506.x

[39] Huse M, Kuriyan J (2002) The conformational plasticity of protein kinases. Cell 109: 275–282. 12015977 10.1016/s0092-8674(02)00741-9

[40] Pereira SF, Goss L, Dworkin J (2011) Eukaryote-like serine/threonine kinases and phosphatases in bacteria. Microbiol Mol Biol Rev 75: 192–212. 10.1128/MMBR.00042-10 21372323 PMC3063355

[41] Chandra G, Chater KF (2014) Developmental biology of *Streptomyces* from the perspective of 100 actinobacterial genome sequences. FEMS Microbiol Rev 38: 345–379. 10.1111/1574-6976.12047 24164321 PMC4255298

[42] Jakimowicz D, van Wezel GP (2012) Cell division and DNA segregation in *Streptomyces*: how to build a septum in the middle of nowhere? Mol Microbiol 85: 393–404. 10.1111/j.1365-2958.2012.08107.x 22646484

[43] Noens EE, Mersinias V, Traag BA, Smith CP, Koerten HK, van Wezel GP (2005) SsgA-like proteins determine the fate of peptidoglycan during sporulation of *Streptomyces coelicolor* . Mol Microbiol 58: 929–944. 16262781 10.1111/j.1365-2958.2005.04883.x

[44] Grantcharova N, Ubhayasekera W, Mowbray SL, McCormick JR, Flardh K (2003) A missense mutation in *ftsZ* differentially affects vegetative and developmentally controlled cell division in *Streptomyces coelicolor* A3(2). Mol Microbiol 47: 645–656. 12535067 10.1046/j.1365-2958.2003.03334.x

[45] Molle V, Kremer L (2010) Division and cell envelope regulation by Ser/Thr phosphorylation: *Mycobacterium* shows the way. Mol Microbiol 75: 1064–1077. 10.1111/j.1365-2958.2009.07041.x 20487298

[46] Thakur M, Chakraborti PK (2006) GTPase activity of mycobacterial FtsZ is impaired due to its transphosphorylation by the eukaryotic-type Ser/Thr kinase, PknA. J Biol Chem 281: 40107–40113. 17068335 10.1074/jbc.M607216200

[47] Thakur M, Chakraborti PK (2008) Ability of PknA, a mycobacterial eukaryotic-type serine/threonine kinase, to transphosphorylate MurD, a ligase involved in the process of peptidoglycan biosynthesis. Biochem J 415: 27–33. 10.1042/BJ20080234 18557704

[48] Kang CM, Abbott DW, Park ST, Dascher CC, Cantley LC, Husson RN (2005) The *Mycobacterium tuberculosis* serine/threonine kinases PknA and PknB: substrate identification and regulation of cell shape. Genes Dev 19: 1692–1704. 15985609 10.1101/gad.1311105 PMC1176007

[49] Soares NC, Spat P, Krug K, Macek B (2013) Global dynamics of the *Escherichia coli* proteome and phosphoproteome during growth in minimal medium. J Proteome Res 12: 2611–2621. 10.1021/pr3011843 23590516

[50] Ravikumar V, Shi L, Krug K, Derouiche A, Jers C, Cousin C, et al (2014) Quantitative phosphoproteome analysis of *Bacillus subtilis* reveals novel substrates of the kinase PrkC and phosphatase PrpC. Mol Cell Proteomics 13: 1965–1978. 10.1074/mcp.M113.035949 24390483 PMC4125730

[51] Manteca A, Ye J, Sanchez J, Jensen ON (2011) Phosphoproteome analysis of *Streptomyces* development reveals extensive protein phosphorylation accompanying bacterial differentiation. J Proteome Res 10: 5481–5492. 10.1021/pr200762y 21999169

[52] Parker JL, Jones AM, Serazetdinova L, Saalbach G, Bibb MJ, Naldrett MJ, (2010) Analysis of the phosphoproteome of the multicellular bacterium *Streptomyces coelicolor* A3(2) by protein/peptide fractionation, phosphopeptide enrichment and high-accuracy mass spectrometry. Proteomics 10: 2486–2497. 10.1002/pmic.201000090 20432484

[53] Yague P, Rodriguez-Garcia A, Lopez-Garcia MT, Martin JF, Rioseras B, Sanchez J, et al (2013) Transcriptomic analysis of *Streptomyces coelicolor* differentiation in solid sporulating cultures: first compartmentalized and second multinucleated mycelia have different and distinctive transcriptomes. PLoS One 8: e60665 10.1371/journal.pone.0060665 23555999 PMC3610822

[54] Del Sol R, Mullins JG, Grantcharova N, Flardh K, Dyson P (2006) Influence of CrgA on assembly of the cell division protein FtsZ during development of *Streptomyces coelicolor* . J Bacteriol 188: 1540–1550. 16452438 10.1128/JB.188.4.1540-1550.2006 PMC1367258

[55] Eccleston M, Willems A, Beveridge A, Nodwell JR (2006) Critical residues and novel effects of overexpression of the *Streptomyces coelicolor* developmental protein BldB: evidence for a critical interacting partner. J Bacteriol 188: 8189–8195. 16963568 10.1128/JB.01119-06 PMC1698190

[56] Nariya H, Inouye S (2005) Identification of a protein Ser/Thr kinase cascade that regulates essential transcriptional activators in *Myxococcus xanthus* development. Mol Microbiol 58: 367–379. 16194226 10.1111/j.1365-2958.2005.04826.x

[57] Petrickova K, Tichy P, Petricek M (2000) Cloning and characterization of the pknA gene from *Streptomyces coelicolor* A3(2), coding for the Mn2+ dependent protein Ser/Thr kinase. Biochem Biophys Res Commun 279: 942–948. 11162454 10.1006/bbrc.2000.4054

[58] Sambrook JM T.; Russel D.W. (2001) Molecular cloning: a laboratory manual: Cold Spring Harbor Laboratory Press.

[59] Kieser T, Bibb MJ, Buttner MJ, Chater KF, Hopwood DA (2000) Practical *Streptomyces* Genetics. Norwich, UK: The John Innes Foundation.

[60] Flett F, Mersinias V, Smith CP (1997) High efficiency intergeneric conjugal transfer of plasmid DNA from *Escherichia coli* to methyl DNA-restricting streptomycetes. FEMS Microbiol Lett 155: 223–229. 9351205 10.1111/j.1574-6968.1997.tb13882.x

[61] Molle V, Leiba J, Zanella-Cleon I, Becchi M, Kremer L (2010) An improved method to unravel phosphoacceptors in Ser/Thr protein kinase-phosphorylated substrates. Proteomics 10: 3910–3915. 10.1002/pmic.201000316 20925060

[62] Borchert N, Dieterich C, Krug K, Schutz W, Jung S, Nordheim A, et al (2010) Proteogenomics of *Pristionchus pacificus* reveals distinct proteome structure of nematode models. Genome Res 20: 837–846. 10.1101/gr.103119.109 20237107 PMC2877580

[63] Conzelmann M, Williams EA, Krug K, Franz-Wachtel M, Macek B, Jekely G, (2013) The neuropeptide complement of the marine annelid *Platynereis dumerilii* . BMC Genomics 14: 906 10.1186/1471-2164-14-906 24359412 PMC3890597

[64] Cox J, Mann M (2008) MaxQuant enables high peptide identification rates, individualized p.p.b.-range mass accuracies and proteome-wide protein quantification. Nat Biotechnol 26: 1367–1372. 10.1038/nbt.1511 19029910

[65] Cox J, Neuhauser N, Michalski A, Scheltema RA, Olsen JV, Mann M,. (2011) Andromeda: a peptide search engine integrated into the MaxQuant environment. J Proteome Res 10: 1794–1805. 10.1021/pr101065j 21254760

[66] Karimova G, Pidoux J, Ullmann A, Ladant D (1998) A bacterial two-hybrid system based on a reconstituted signal transduction pathway. Proc Natl Acad Sci U S A 95: 5752–5756. 9576956 10.1073/pnas.95.10.5752 PMC20451

