# Supplementary material for: Control of Morphological Differentiation of Streptomyces coelicolor A3(2) by Phosphorylation of MreC and PBP2
Source: PLoS One. 2015 Apr 30;10(4):e0125425. doi: 10.1371/journal.pone.0125425 (PMC4416010; doi:10.1371/journal.pone.0125425)
Supplement: S1 Table — (DOCX) [file pone.0125425.s007.docx]

**Table S1. Plasmids used in this study**

| **Name** | **Characteristics** | **Source** |
| --- | --- | --- |
| pCDF-Duet1 | 2x P_T7_, *aad,* simultaneous expression of two proteins | Novagen |
| pKT18 | Cloning vector, *aphII* | Pridmore, 1987 |
| pGUS21 | Cloning vector, *aac(3)IV, gusA* | Muth, unpublished |
| pKO4778 | pKT18 derivative, deletion vector for *SCO4778*, *aphII* | Present study |
| pGus-KO4775-4779 | pGus21 derivative, deletion vector for *SCO4775-4779, aac(3)IV* | Present study |
| pSET152 | Cloning vector, *aac(3)IV,* integrative, ΦC31 *attP* | Bierman et al., 1992 |
| pSET-pkaH | *pkaH* complementation plasmid | Present study |
| pSET-4776 | *SCO4776* complementation plasmid | Present study |
| pSET-pkaD | *pkaD* complementation plasmid | Present study |
| pSET-pkaI | *pkaI* complementation plasmid | Present study |
| pSET-pkaJ | *pkaJ* complementation plasmid | Present study |
| pCDF-PkaI | *pkaI* N-terminal His-tag | Present study |
| pCDF-PkaI-MreC | *pkaI* N-terminal His-tag,  *mreC* C-terminal S-tag | Present study |
| pCDF-MreC | *mreC* C-terminal S-tag | Present study |
| pCDF-PBP2 | *pbp2* C-terminal S-tag | Present study |
| pCDF-PkaI-PBP2 | *pkaI* N -terminal His-tag,  *pbp2* C -terminal S-tag | Present study |
| pYT9-Crp | STSU_15619 n-terminal Strep-tag II,  P_Rham_, *bla* | S. Kocadinc, pers. communication |
| pUT18c | *bla, cya*-T18 | Karimova et al., 1998 |
| pUT18c-4778 | *bla, cya*-T18, *SCO4778* | Present study |
| pUT18c-4778_257-380_ | *bla, cya*-T18, *SCO4778*_257-380_ | Present study |

**Bierman M, Logan R, O`Brien K, Seno ET, Nagaraja-Rao R, Schoner BE.** 1992. Plasmid cloning vectors for the conjugal transfer of DNA from *Escherichia coli* to *Streptomyces* spp. Gene ***116***: 43-49.

**Pridmore RD.** 1987. New and versatile cloning vectors with kanamycin-resistance marker. Gene ***56***, 309-312.

**Karimova G, Pidoux J, Ullmann A, Ladant D**. 1998. A bacterial two-hybrid system based on a reconstituted signal transduction pathway. Proc Natl Acad Sci U S A 95: 5752-5756.
