# Supplementary material for: Control of Morphological Differentiation of Streptomyces coelicolor A3(2) by Phosphorylation of MreC and PBP2
Source: PLoS One. 2015 Apr 30;10(4):e0125425. doi: 10.1371/journal.pone.0125425 (PMC4416010; doi:10.1371/journal.pone.0125425)
Supplement: S2 Table — (DOCX) [file pone.0125425.s008.docx]

**Table S2. Primers* used in this study**

| **Name** | **Sequence** | **Application** |
| --- | --- | --- |
| Intern4778f | TCATCGTCGTGCACGA | Control *pkaI* deletion |
| Intern4778r | ATCTCCTCGGTGACCA |  |
| Up4778K416 | CTCTACCTGCCCGAGAACTG | Control *pkaI* deletion |
| Lo4778K2240 | GGTTGCAGGTCAAAACTCGT |  |
| Up4778fwE | CCG*AATTC*GATTGTGATGGAGCTG | *pkaI* deletion |
| Up4778revB | AA*GGATCC*CATCATGCCCCACCGG |  |
| lo4778fwB | GC*GGATCC*TAAACGGGAGATAAGC |  |
| lo4778revH | GG*AAGCTT*AGACGACCGTGTTCTC |  |
| c4775-79fw | TCGGGTGAGGGACAATGAAG | Control *SCO4775-4779* deletion |
| c4775-79rev | AGCACGACCTGGAAGACCTC |  |
| Up4775Ef | AA*GAATTC*GGACTGCTTCTGGAAC | *SCO4775-4779* deletion |
| Up4775Br | CC*GGATCC*CATGCGTCCCCTCAT |  |
| Lo4779Bf | GG*GGATCC*AATCTGGACATCGACG |  |
| Lo4779Hr | AA*AAGCTT*CTCGTTGATGTTGACG |  |
| 4778DuUpBg | CC*AGATCT*GATGGGGCGCATGGTGA | Expression of PkaI |
| 4778DuLoH | GG*AAGCTT*GACGCACGATGCCGC |  |
| PBP2DuUpN | AA*CATATG*ACCAACATCCCCGAGAC | Expression of PBP2 |
| PBP2DuLoX | AA*CTCGAG*TACGCGTGCCCTCCG |  |
| MreCDuUpN | GG*CATATG*AGGGACACGAAAGAGAGC | Expression of MreC |
| MreCDuLoK | AA*GGTACC*CGGCTCGGGCTGCTCGTTCT |  |
| pSet4775fwXbaI | GG*TCTAGA*GAGATCGAGCACTACGAGAAG | Expression of *pkaH* in M145  and NLΔ4775-4779 |
| pSet4775revEco | TT*GAATTC*CCGCAACCACTCACGATAC |  |
| pSet4776fwXbaI | GG*TCTAGA*CTTCGAACCCAAGTCCTGAG | Expression of *SCO4776* in M145 and NLΔ4775-4779 |
| pSet4776revEco | TT*GAATTC*CTTGTCAGGGTCAGACTAGG |  |
| pSet4777fwXbaI | GG*TCTAGA*TCGGCCACCTACCAGTACAC | Expression of *pkaD* in M145 and NLΔ4775-4779 |
| pSet4777revEco | TT*GAATTC*GTTCAGCCGGTCTCCTCCT |  |
| Up4778XSet | GG*TCTAGA*GTACGCCCTCAACATGAC | Expression of *pkaI* in M145, Complementation of NLΔPkaI |
| Lo4778ESet | GG*GAATTC*ATGCCGCTTATCTCCC |  |
| pSet4779fwXbaI | GG*TCTAGA*CGGTACGCCGACCAGTTC | Expression of *pkaJ* in M145 and NLΔ4775-4779 |
| pSet4779revEco | TT*GAATTC*TGAGCCGCAGGGTATGAC |  |
| RT4775fw | ACATCCCGTACGGACAAGAG | RT-PCR |
| RT4775re | GACGAGTTCCATCACGATCC |  |
| RT4776fw | CGAGTACGAACTCACCGAGA | RT-PCR |
| RT4776re | CGAAGACGTCGAAGACCTG |  |
| RT4777fw | GGATTGTGATGGAGCTGGTC | RT-PCR |
| RT4777re | GATGCCGAAGTCGGTGAG |  |
| RT4778fw | GCGTACATGGTCATGGAACT | RT-PCR |
| RT4778re | GGTGTACTCGGGGGAACC |  |
| RT4779fw | TGGATCGTGATGGAACTCGT | RT-PCR |
| RT4779re | GTGTCGGTCAGGCTGGTC |  |
| RT-in75+76.fw | CTTCGAACCCAAGTCCTGAG | RT-PCR |
| RT-in75+76.rev | GGGTCTCGGTGAGTTCGTA |  |
| RT-in76+77.fw | TCGGCCACCTACCAGTACAC | RT-PCR |
| RT-in76+77.rev | GCGGTCAACTCCTTGATGG |  |
| RT-in77+78.fw | GTCGAGTACGCCCTCAACAT | RT-PCR |
| RT-in77+78.rev | GCTCATGGAGCCGGTAAC |  |
| RT-in78+79.fw | GGCACCTAAACGGGAGATAA | RT-PCR |
| RT-in78+79.rev | GTACGACCTGGGTCGGTGT |  |
| 2H4778fwX | AC*TCTAGA*GCGCATGGTGACCGAG | BTH |
| 2H4778revE | AT*GAATTC*CCGTTTAGGTGCCGT | BTH |
| 2H4778.257fwX | AC*TCTAGA*CCTGCTGGAGCGCGATC | BTH |

*Restriction sites indicated in italics
