# Supplementary material for: Control of Morphological Differentiation of Streptomyces coelicolor A3(2) by Phosphorylation of MreC and PBP2
Source: PLoS One. 2015 Apr 30;10(4):e0125425. doi: 10.1371/journal.pone.0125425 (PMC4416010; doi:10.1371/journal.pone.0125425)
Supplement: S5 Table — (DOCX) [file pone.0125425.s011.docx]

**Table S5. *S. coelicolor* interaction partners of the eSTPK PkaI**

| **Category** | **Protein** | **Times found/ library^§^** | **Putative function/ characteristics** |
| --- | --- | --- | --- |
| **Differentiation** | SCO1403* | 5 E | putative membrane protein, interacts with MreC and RodZ |
|  | SCO2097* | 35 E | Actinomycetes signature protein, part of *dcw* cluster, role in sporulation, interacts with MreC, MreD, PBP2, Sfr, FtsI, RodZ |
|  | SCO2897 | 1 N | probable secreted penicillin-binding protein |
|  | SCO3110* | 1 N | FtsX-like ABC transporter int. membrane subunit, interacts with MreC |
|  | SCO3754* | 1 N | FtsX-like ABC transporter int. membrane subunit interacts with MreC, MreD |
|  | SCO3854 (CrgA) | 1 N | Septation inhibitor protein, coordinates growth and cell division in aerial hyphae |
|  | SCO4907(AfsQ1) | 1 N | pleiotropic transcriptional regulator |
|  | SCO5587 (FtsH) | 1 N | cell division protein, zinc metalloprotease |
|  | SCO5723 (BldB) | 1 N | transcriptional regulator, differentiation |
|  | SCO6494* | 2 E | put. membrane protein, interacts with MreD |
| **eSTPK** | SCO2974 (PkaA) | 2 N | serine/threonine protein kinase |
|  | SCO4777 (PkaD) | 2 N | serine/threonine protein kinase |
|  | SCO4778* (PkaI) | 1 E | serine/threonine protein kinase, interacts with MreC, MreD, PBP2, Sfr, FtsI, RodZ |
|  | SCO7326 | 1 E | serine/threonine phosphatase |
| **Other** | SCO1058 (UgpE) | 1 N | ABC transporter, transmembrane subunit |
|  | SCO4140 (PstA) | 1 N | phosphate ABC transport system permease |
|  | SCO6374 | 1 N | putative sugar transferase |
| **Hypothetical**  **proteins** | SCO1465 | 1 E | putative secreted protein |
|  | SCO1829 | 2 N | putative membrane protein |
|  | SCO2169 | 2 N | putative integral membrane protein |
|  | SCO2255 | 1 N | putative membrane protein |
|  | SCO3146 | 1 N | putative secreted protein |
|  | SCO3218 | 1 N | conserved hypothetical protein |
|  | SCO3855 | 1 E | putative membrane protein |
|  | SCO3940 | 1 N | putative transmembrane protein |
|  | SCO4431 | 3 N | putative integral membrane protein |
|  | SCO4641 | 1 N | putative transmembrane efflux protein |
|  | SCO4846 | 1 E | putative integral membrane protein |
|  | SCO5013 | 3 N | putative secreted protein |
|  | SCO5148 | 1 N | putative membrane protein |
|  | SCO5485 | 1 N | small hydrophobic membrane protein |
|  | SCO5587 | 1 N | large hypothetical protein |
|  | SCO5935 | 1 N | putative membrane protein |
|  | SCO5993 | 1 N | putative membrane protein |
|  | SCO6074 | 1 E | putative integral membrane protein |
|  | SCO6614 | 1 N | putative membrane protein |
|  | SCO6899 | 1 E | putative membrane protein |
|  | SCO7482 | 1 E | putative integral membrane protein |

^§^ E: enzymatically generated library; N: nebulized library

* Previously identified as components of the SSSC (Kleinschnitz et al., 2011)
